# 

**Published:** 1890-08-02

**Authors:** 


### SATURDAY, AUGUST 2nd, 1890.

**Fair Play** - 257
**Words of Consolation.**—Submission - 258
**The Doctor's Pulpit.**—Baby Brains - 259
**Turning Over New Leaves.**—Faith Cures - 260
**Before the Lords' Committee.** IX.—Hospital and District Work - 261
**The Metropolitan Hospital Sunday Fund, 1890** - 262
**Everybody's Page** - 263
**Notes and News** - 264
**Annotations.**—Lady Novelists and Tall Men—Pasteurism and Politeness—Hume and Miracles - 266
**Books Received** - 271
**The Practitioner's Mirror and Retrospect:—**
Medicine—Nocturnal Incontinence of Children—Recent Research - 267
Surgery—Nephrolithotomy - 267
The Practitioner's Bookshelf—The Life and Works of Harvey - 269
New Drugs, Appliances, and Things Medical—Meeting of the British Medical Association at Birmingham - 270
**The Neglect of the Poor in Workhouse Infirmaries.** By Louisa Twining - 270
**Passing Topics**—Trivialities - 272
**Scraps and Gleanings** - 272

### EXTRA SUPPLEMENT—THE NURSING MIRROR.

**En Passant.**—A New Home at Brighton—A Prayer Society—Short Items—The Eight Hours Day—Help Wanted—Kent Nursing Institution—Paris Institutions—Nurses—British Nurses' Association - lxxv
**Sketches of Insanity.**—By Francis H. Walmsley, M.D. V.—Mania - lxxvi
**Dr. Tarrant's Mistake** - lxxvi
**Everybody's Opinion.**—Homes for Epileptics—Nurses in Asylums, lxxvii.; Loyalty—Charity Organisation Society lxxviii
**Notes and Queries** - lxxviii
**Appointments—Vacancies** - lxxviii
**Amusements and Relaxation** - lxxviii

